# MHD Flow of Sodium Alginate-Based Casson Type Nanofluid Passing Through A Porous Medium With Newtonian Heating

**DOI:** 10.1038/s41598-018-26994-1

**Published:** 2018-06-05

**Authors:** Arshad Khan, Dolat Khan, Ilyas Khan, Farhad Ali, Faizan ul Karim, Muhammad Imran

**Affiliations:** 1 Institute of Business and Management Sciences, The University of Agriculture, Peshawar, Khyber Pakhtunkhwa Pakistan; 2 Department of Mathematics, City University of Science and Information Technology, Peshawar, 25000 Pakistan; 3 Basic Engineering Sciences Department, College of Engineering Majmaah University, Majmaah, 11952 Saudi Arabia; 4 Computational Analysis Research Group, Ton Duc Thang University, Ho Chi Minh City, Vietnam; 5 Department of Mathematical Sciences, United Arab Emirates University, P. O. Box, 15551 Al Ain, United Arab Emirates; 6 Faculty of Mathematics and Statistics, Ton Duc Thang University, Ho Chi Minh City, Vietnam

## Abstract

Casson nanofluid, unsteady flow over an isothermal vertical plate with Newtonian heating (NH) is investigated. Sodium alginate (base fluid)is taken as counter example of Casson fluid. MHD and porosity effects are considered. Effects of thermal radiation along with heat generation are examined. Sodium alginate with Silver, Titanium oxide, Copper and Aluminum oxide are added as nano particles. Initial value problem with physical boundary condition is solved by using Laplace transform method. Exact results are obtained for temperature and velocity fields. Skin-friction and Nusselt number are calculated. The obtained results are analyzed graphically for emerging flow parameters and discussed. It is bring into being that temperature and velocity profile are decreasing with increasing nano particles volume fraction.

## Introduction

The fluid is a particular kind of matter which have no fixed shape and deforms easily due to external pressure^1^. Fluids are mainly of two type’s i.e Newtonian and non-Newtonian. Non-Newtonian fluids have numerous industrial applications^2,3^. Furthermore, its application with magnetohydrodynamic (MHD) flow in a porous medium can widely be seen in irrigation problem, biological system, petroleum, textile, polymer industries. More investigations have been published on numerous aspects of MHD non-Newtonian fluid passes over a porous medium^4–7^. The entropy analysis for nanofluid with different type of nano particles and water type base fluid for unsteady MHD flow was studied by^8^. The impact of magnetic field on free convection of nanofluid in a porous medium is presented by^9^. The effects of heat transfer on MHD nanofluid in a porous semi annulus has investigated by^10^ using numerical methods. Sheikholeslami *et al*.^11^ examined the influence of free convection in a semi annulus enclosure for ferrofluid flow in the presence of magnetic source with the consideration of thermal radiation. The observation of non-uniform magnetic field and variable magnetic field on forced convection heat is investigated by^12,13^. The observation of MHD on fluid flow with heat transfer is studded by^14–16^. Recently^17,18^ investigated the nanofluid transportation in a in the presence of magnetic source and porous cavity using *CuO* nano particles. The influence of external magnetic field for nanofluid as water is a base fluid of free convection flow is studied in^19^. Sheikholeslami and Ganji^20^ have investigated the effect of convective heat transfer for the nanofluid by semi analytical and numerical approaches. The same author has also investigated the influence of heat transfer for nanofluid between parallel plates in^21^. The influence of Lorentz forces and convection nanofluid flow is investigated by^22–24^. Dissimilar types of nano particles with water based fluid are studied by^25,26^. The influence of melting heat for nanofluid is studied by^27^. The transportation of nanofluid in porous media is investigated by^28^. The influence of magnetic field for nanofluid with entropy generation is analysed by^29–31^.

Nanotechnology is that kind of technology which provides the materials with size less than $100$ nm called nanomaterials. On the basis of the structure and their properties, nanomaterials are divided into four categories^32^. Carbon based nano materials, metal based nano materials, Dendrimers and composite. The terminology of nanofluid was first investigated by Choi^33^. He defined that the fluids occupying the sizes of particles less than 100 nm is called nanofluid. The categorieswith different attitude of nano particles are particle material, Base fluid, size and concentration, of the nanofluid. Suspend these nano particles into any type of conventional fluid like oil, water, ethylene glycol to make nanofluids. The reason why nano size particles are preferred over micro size particles has been explained by^34^. Nano particles over micro particles, good improvement have seen in thermo physical properties. Nanofluids have various applications such as in air conditioning cooling, automotive, power plant cooling, improving diesel generator efficiency etc.^35^. Usually water, ethylene glycol are utilized as heat transfer base fluids. Different substances are used for the production of nanoparticles, which are generally divided into metallic i.e. copper^36^, metal-oxide i.e. CuO^37^, chalcogenides sulphides, selenides and telluride’s, mentioned^38^ and different particles, such like carbon nanotubes^39^. In literature the size of one particle is in between 20 nm^40^ and 100 nm^41^.

Casson fluid model was first presented by Casson in 1959. Casson fluids in tubes was first studied by Oka^42^. Examples of Casson fluids are honey, blood, soup, jelly, stuffs, slurries, artificial fibers etc. Cassonnanofluid flow with Newtonian heatingpresented by^43^. Sarojamma *et al*.^44^ investigated Casson nanofluid past over perpendicular cylinder in the occurrence of a transverse magnetic field with internal heat generation or absorption.

Khalid *et al*.^45^ examined unsteady MHD Casson fluid withfree convection flow in a porous medium. Bhattacharyya *et al*.^46^ studied systematically magnetohydrodynamic Casson fluid flow over a stretching shrinking sheet with wall mass transfer. Arthur *et al*.^47^ studied Casson fluid flow in excess of a perpendicular porous surface, chemical reaction in the existence of magnetic field. Recently, Fetecau *et al*.^48^ has investigated fractional nanofluids for natural convection flow over an isothermal perpendicular plate with thermal radiation. Hussanan *et al*.^49^ investigates the unsteady heat transfer flow of a non-Newtonian Casson fluid over an oscillating perpendicular plate with Newtonian heating. Recently, Imran *et al*.^50^ analyzed the effect of Newtonian heating with slip condition on MHD flow of Casson fluid. MHD flow of Casson fluid with heat transfer and Newtonian heating is analyzed by Hussanan *et al*.^51^. The effect of Newtonian heating for nanofluid is recently investigated by^43,52^. But no work is done until now on heat transfer enhancement in Sodium alginate fluid with additional effects of NH, MHD, porosity, heat generation, and thermal radiation. Silver (*Ag*), Titanium oxide (*TiO*_2_), Copper (*Cu*) and Aluminum oxide (*Al*_2_*O*_3_) are nano particles suspended in base fluid. Problem is solved and interpreted graphically with some conclusions.

## Mathematical Modeling and solution of the Problem

Sodium alginate with Silver (*Ag*), Titanium oxide (*TiO*_2_), Copper (*Cu*) and Aluminum oxide (*Al*_2_*O*_3_) nano particles is considered. Heat transfer, thermal radiation and heat generation are taken. Unsteady flow is over an infinite vertical plate (*ξ* > 0) embedded in a saturated porous medium. MHD effect with uniform magnetic field *B* of strength *B*_0_ and small magnetic Reynolds number. Initially both the plate and fluid are at rest with constant temperature Θ_∞_. At time *t* = 0^+^ the plate originates oscillation in its plane *ξ* = 0 according to condition

1 $$u=UH(t)\cos (\omega t)i;\,{\rm{or}}\,{\rm{u}}=U\,\sin (\omega t)i;\,t > 0$$

After some time, plate temperature is raised to Θ_*w*_. The fluid is electrically conducting. Therefore, by Maxwell equations

2 $${\rm{div}}{\bf{B}}={\bf{0}},\,{\rm{Curl}}{\bf{E}}={\boldsymbol{-}}\frac{\partial {\bf{B}}}{\partial t},\,{\rm{Curl}}{\bf{B}}={\mu }_{e}{\bf{J}}.$$

By using Ohm’s law

3 $${\bf{J}}={\sigma }_{nf}({\bf{E}}{\boldsymbol{+}}{\bf{V}}{\boldsymbol{\times }}{\bf{B}}),$$

The quantities *ρ*_*nf*_, *μ*_*e*_ and *σ* are assumed constants. Magnetic field **B** is normal to **V**. The Reynolds number is so small that flow is laminar. Hence,

4 $$\frac{1}{{\rho }_{nf}}{\bf{J}}\times {\bf{B}}=\frac{{\sigma }_{nf}}{{\rho }_{nf}}[({\bf{V}}\times {{\bf{B}}}_{0})\times {{\bf{B}}}_{0}]=-\frac{{\sigma }_{nf}{B}_{0}^{2}{\bf{V}}}{{\rho }_{nf}}.$$

Equation for an incompressible Casson fluid flow^53–55^

5 $$\tau ={\tau }_{0}+\mu {\gamma }^{\bullet }$$

Or

6 $${\tau }_{ab}=\{\begin{array}{cc}2({\mu }_{\eta }+\frac{{p}_{\lambda }}{\sqrt{2\pi }}){e}_{ab}, & \pi  > {\pi }_{c}\\ 2({\mu }_{\eta }+\frac{{p}_{\lambda }}{\sqrt{2{\pi }_{c}}}){e}_{ab}, & \pi  > {\pi }_{c}\end{array},$$

where *π* = *e*_*ab*_*e*_*ab*_ and *e*_*ab*_ is the (*a*, *b*)^*ah*^ factor of the deformation rate, *π* is represent the product of the factor of deformation rate with itself, *π*_*c*_ is represent the critical value of this product based on the non-Newtonian model, *μ*_*η*_ is represent the plastic dynamic viscosity of the non- Newtonian fluid and *P*_*λ*_ is yield stress of fluid. Under these conditions alongside with the assumption that the viscous dissipation term in the energy equation is neglected, we get the following system^56^:

7 $${\rho }_{nf}({u}_{t})=\,(1+\frac{1}{\gamma }){\mu }_{nf}({u}_{\xi \xi })-({\sigma }_{nf}{B}_{0}^{2}+(1+\frac{1}{\gamma })\frac{{\mu }_{nf}\psi }{k})u+g{(\rho \beta )}_{nf}[{\rm{\Theta }}-{{\rm{\Theta }}}_{\infty }];\,t,\,\xi  > 0,$$

8 $${(\rho {c}_{p})}_{nf}{{\rm{\Theta }}}_{t}={k}_{nf}(1+\frac{16{\sigma }^{\ast }{{\rm{\Theta }}}_{\infty }^{3}}{3{k}_{nf}{k}^{\ast }}){T}_{\xi \xi }+{Q}_{0}({\rm{\Theta }}-{{\rm{\Theta }}}_{\infty });\,\xi ,\,t > 0,$$

9 $$\begin{array}{c}u=0,\,{\rm{\Theta }}={{\rm{\Theta }}}_{\infty };\,\xi \ge 0,\,t < 0\\ u=UH(t)\,\cos (\omega t)\,{\rm{or}}\,u=U\,\sin (\omega t),\,\frac{\partial {\rm{\Theta }}}{\partial \xi }=-{h}_{s}{\rm{\Theta }};\,t\ge 0,\,\xi =0\\ u\to 0,\,{\rm{\Theta }}\to {{\rm{\Theta }}}_{\infty }\,{\rm{as}}\,\xi \to \infty \end{array}\},$$

where k^*^ is absorption coefficient and σ^*^ is Stefan-Boltzmann constant. Where *Q*_0_ is the heat generation term, *ρ*_*nf*_ is the density of nanofluids, *μ*_*nf*_ is the dynamic viscosity, *u* is the fluid velocity in the $x$-axis perpendicular direction, *γ* is the Casson fluid parameter, *ψ*(0 < *ψ* < 1), *K* > 0, *ψ* is the porous medium and *K* is the permeability of porous medium, *h*_*s*_ is a constant heat transfer coefficient, Θ_*w*_ is the constant plate temperature (Θ_*w*_ < Θ_∞_, Θ_*w*_ > Θ_∞_ due to the cooled or heated plate, respectively), *g* is the acceleration due to gravity, and *β*_*nf*_ is the thermal expansion coefficient of the nanofluid.

Expressions for (*ρc*_*p*_)_*nf*_, (*ρβ*)_*nf*_, *μ*_*nf*_, *ρ*_*nf*_, *σ*_*nf*_, *k*_*nf*_ are given by^24^:

10 $$\begin{array}{rcl}{\rho }_{nf} & = & (1-\varphi ){\rho }_{f}+\varphi {\rho }_{s},\,{\mu }_{nf}=\frac{{\mu }_{f}}{{(1-\varphi )}^{2.5}},\,\sigma =\frac{{\sigma }_{f}}{{\sigma }_{s}},\\ {(\rho \beta )}_{nf} & = & (1-\varphi ){(\rho \beta )}_{f}+\varphi {(\rho \beta )}_{s},\,{(\rho {c}_{p})}_{nf}=(1-\varphi ){(\rho {c}_{p})}_{f}+\varphi {(\rho {c}_{p})}_{s},\\ {k}_{nf} & = & {k}_{f}(\frac{{k}_{s}+2{k}_{f}-2\varphi ({k}_{f}-{k}_{s})}{{k}_{s}+2{k}_{f}+\varphi ({k}_{f}-{k}_{s})}),\,{\sigma }_{nf}={\sigma }_{f}(1+\frac{3(\sigma -1)\varphi }{(\sigma +2)-(\sigma -1)\varphi }),\end{array}$$

where *ϕ* the volume fraction of nano particles, *ρ*_*f*_ and *ρ*_*s*_ is represent the density of base fluid and particle respectively, and *c*_*p*_ is specific heat on constant pressure. *k*_*nf*_, *k*_*f*_, and *k*_*s*_ are the thermal conductivities of the nanofluid, the base-fluid, and the solid particles, respectively. The expressions of Eq. (10) are classified to nano particles^57^. For supplementary nano particles with unlike thermal conductivity, dynamic viscosity, see to Table 1^58–60^.

**Table 1:** Thermophysical properties of nanofluids^58–60^.

	*ρ* (*kgm*^−3^)	*c*_*p*_ (*kg*^−1^*k*^−1^)	*k* (*Wm*^−1^*k*^−1^)	*β* × 10^−5^ (*k*^−1^)
C_6_H_9_NaO_7_(*SA*)	989	4175	0.613	0.99
*Al*_2_ *O*_3_	3970	765	40	0.85
*Cu*	8933	385	401	1.67
*TiO*_2_	4250	686.2	8.9528	0.9
*Ag*	10500	235	429	1.89

the dimensionless variables are^56^,

11 $$[{u}^{\ast }=\frac{u}{{U}_{0}},\,{\xi }^{\ast }=\frac{{U}_{0}}{\nu }\xi ,\,{t}^{\ast }=\frac{{{U}_{0}}^{2}}{\nu }t,\,{\theta }=\frac{{\rm{\Theta }}-{{\rm{\Theta }}}_{\infty }}{{{\rm{\Theta }}}_{w}-{{\rm{\Theta }}}_{\infty }}],$$

Into Eqs (7–9), we get

12 $${u}_{t}={c}_{2}{u}_{\xi \xi }-Hu+G{r}_{0}{\theta }\,t,\,\xi  > 0,$$

13 $${{\theta }}_{t}={c}_{4}{{\theta }}_{\xi \xi }+{c}_{5}{\theta };\,\xi ,\,t > 0$$

14 $$\begin{array}{c}u=0,\,{\theta }=0;\,\xi \ge 0,\,t < 0\\ u=H(t)\cos (\omega t)\,{\rm{or}}\,u=\,\sin (\omega t),\,{{\theta }}_{\xi }=-\lambda (1+{\theta });\,t\ge 0,\,\xi =0\\ u\to 0,\,{\theta }\to 0\,{\rm{as}}\,\xi \to \infty \end{array}\}$$

where

$$\begin{array}{rcl}{\phi }_{1} & = & {(1-\varphi )}^{2.5}[1-\varphi +\varphi (\tfrac{{\rho }_{s}}{{\rho }_{f}})],\,{c}_{1}=1+\tfrac{3(\sigma -1)\varphi }{(\sigma +2)-(\sigma -1)\varphi },\,{c}_{2}=(1+\tfrac{1}{\gamma })\tfrac{1}{{\phi }_{1}},\\ {c}_{3} & = & 1-\varphi +\varphi \tfrac{{(\rho {c}_{p})}_{s}}{{(\rho {c}_{p})}_{f}},\,{c}_{4}=\tfrac{{\lambda }_{nf}(1+N{r}_{0})}{\nu \,{\rm{\Pr }}\,{c}_{3}},\,{c}_{5}=\tfrac{\nu {Q}_{0}}{{U}_{0}^{2}{k}_{f}},\,{\lambda }_{nf}=\tfrac{{k}_{nf}}{{k}_{f}},\\ M & = & \tfrac{{c}_{1}{\sigma }_{f}\nu {B}_{0}^{2}}{{\rho }_{f}{U}_{0}^{2}},\,Gr=\tfrac{\nu g{(\rho \beta )}_{f}}{{U}_{0}^{3}{\rho }_{f}}{({\rm{\Theta }}}_{w}-{{\rm{\Theta }}}_{\infty }),\,\tfrac{1}{K}=\tfrac{{\nu }_{f}^{2}\psi }{k{U}_{0}^{2}},\,H=\tfrac{M}{{\phi }_{2}}+\tfrac{{c}_{2}}{{\phi }_{1}K},\\ {\phi }_{2} & = & 1-\varphi +\varphi \tfrac{{\rho }_{s}}{{\rho }_{f}},\,Nr=\tfrac{16{\sigma }^{\ast }{{\rm{\Theta }}}_{\infty }^{3}}{3{k}_{f}{k}^{\ast }},\,{\rm{\Pr }}\tfrac{{(\rho {c}_{p})}_{f}}{{k}_{f}},\,\lambda =\tfrac{{h}_{s}\nu }{{U}_{0}},\,G{r}_{0}=\tfrac{{\phi }_{3}}{{\phi }_{2}}\tfrac{\nu g{(\rho \beta )}_{f}}{{U}_{0}^{3}{\rho }_{f}}{({\rm{\Theta }}}_{w}-{{\rm{\Theta }}}_{\infty })\\ {\phi }_{3} & = & 1-\varphi +\varphi \tfrac{{(\rho \beta )}_{s}}{{(\rho \beta )}_{f}},\,N{r}_{0}=\tfrac{{\lambda }_{nf}}{{k}_{nf}}\tfrac{16{\sigma }^{\ast }{{\rm{\Theta }}}_{\infty }^{3}}{3{k}_{f}{k}^{\ast }}\end{array}$$

where $\frac{1}{K}$ is permeability of pours medium, *M* is the magnetic parameter, *Gr* is thermal Grashof number, Pr is Prandtl number, *Nr* is radiation parameter, and *λ* is Newtonian heating parameter.

## Laplace Transform Solution

Laplace transforms of Eqs (12, 13) gives:

15 $${c}_{2}\overline{{u}_{\xi \xi }}-(q+H)\overline{u}=-G{r}_{0}\overline{{\theta }},$$

16 $${c}_{4}\overline{{{\theta }}_{\xi \xi }}-(q-{c}_{5})\overline{{\theta }}=0,$$

17 $$[\begin{array}{c}\overline{u}=0,\,\overline{{\theta }}=0;\,\xi \ge 0,\,q < 0\\ \overline{u}=\frac{\omega }{{q}^{2}+{\omega }^{2}},\,{\overline{{\theta }}}_{\xi }=-\lambda (\frac{1}{q}+\overline{{\theta }});\,q\ge 0,\,\xi =0\\ \overline{u}\to 0,\,\overline{{\theta }}\to 0\,{\rm{as}}\,\xi \to \infty \end{array}]$$

Eq. (16) using Eq. (17) gives:

18 $$\overline{\theta }\,(\xi ,q)=\frac{1}{q}(\frac{\lambda \sqrt{{c}_{4}}}{\sqrt{q-{c}_{5}}-\lambda \sqrt{{c}_{4}}}){e}^{-\xi \frac{\sqrt{q-{c}_{5}}}{\sqrt{{c}_{4}}}}.$$

After taking the inverse Laplace of Eq. (18):

19 $${\theta }(\xi ,t)=\frac{1}{2}\lambda \sqrt{{c}_{4}}{e}^{-\xi \sqrt{\frac{-{c}_{5}}{{c}_{4}}}}{\int }_{0}^{t}[\begin{array}{c}{e}^{{c}_{5}(t-\tau )}\{\frac{1}{\sqrt{\pi }\sqrt{t-\tau }}+\lambda \sqrt{{c}_{4}}{e}^{{{c}_{6}}^{2}(t-\tau )}erfc(-\lambda \sqrt{{c}_{4}}\sqrt{t-\tau })\}\ast \\ \{2-erfc(\frac{2\sqrt{-{c}_{5}{c}_{4}}\tau -\xi }{2\sqrt{{c}_{4}\tau }})+{{\rm{e}}}^{2y\sqrt{\frac{-c5}{c4}}}erfc(\frac{2\sqrt{-{c}_{5}{c}_{4}}\tau +\xi }{2\sqrt{{c}_{4}\tau }})\}\end{array}]d\tau .$$

Solution of Eq. (15) is:

20 $$\overline{u}(\xi ,q)=[\overline{{u}_{a}}(\xi ,q)+\overline{{u}_{b}}(\xi ,q)+\overline{{u}_{c}}(\xi ,q)+\overline{{u}_{d}}(\xi ,q)].$$

Arranging Eq. (20) as:

21 $$\begin{array}{rcl}\overline{{u}_{a}}(\xi ,q) & = & \frac{\omega }{{q}^{2}+{\omega }^{2}}{e}^{-\xi \frac{\sqrt{q+H}}{\sqrt{{c}_{2}}}},\\ \overline{{u}_{b}}(\xi ,q) & = & \frac{A}{q}({e}^{-\xi \frac{\sqrt{q-{c}_{5}}}{\sqrt{{c}_{4}}}}-{e}^{-\xi \frac{\sqrt{q+H}}{\sqrt{{c}_{2}}}}),\\ \overline{{u}_{c}}(\xi ,q) & = & \frac{{B}_{1}}{q+\frac{{c}_{7}}{{c}_{6}}}({e}^{-\xi \frac{\sqrt{q-{c}_{5}}}{\sqrt{{c}_{4}}}}-{e}^{-\xi \frac{\sqrt{q+H}}{\sqrt{{c}_{2}}}}),\\ \overline{{u}_{d}}(\xi ,q) & = & \frac{C}{\sqrt{q-{c}_{5}}-\lambda \sqrt{{c}_{4}}}({e}^{-\xi \frac{\sqrt{q-{c}_{5}}}{\sqrt{{c}_{4}}}}-{e}^{-\xi \frac{\sqrt{q+H}}{\sqrt{{c}_{2}}}}),\end{array}$$

where

$$\begin{array}{rcl}A & = & \tfrac{{c}_{8}}{{c}_{7}(\sqrt{{c}_{5}}-\lambda \sqrt{{c}_{4}})},\,B=\tfrac{{c}_{8}{c}_{6}}{{c}_{7}(\lambda \sqrt{{c}_{4}}-\sqrt{\tfrac{{c}_{7}}{{c}_{6}}-{c}_{5}})},\,C=\tfrac{{c}_{8}}{{\lambda }^{2}{c}_{4}+{c}_{5}\{{c}_{6}({\lambda }^{2}{c}_{4}+{c}_{5})+{c}_{7}\}},\\ {B}_{1} & = & \frac{B}{{c}_{6}},\,{c}_{6}={c}_{4}-{c}_{2},\,{c}_{7}={c}_{4}H+{c}_{2}{c}_{5},\,{c}_{8}={c}_{4}G{r}_{0}\lambda \sqrt{{c}_{4}},\end{array}$$

Upon inversion:

22 $$u(\xi ,t)=[{u}_{a}(\xi ,t)+{u}_{b}(\xi ,t)+{u}_{c}(\xi ,t)+{u}_{d}(\xi ,t)],$$

where

23 $$\begin{array}{rcl}{u}_{a}(\xi ,t) & = & \frac{1}{4{\rm{i}}}{{\rm{e}}}^{{\rm{i}}tw}[{{\rm{e}}}^{-\xi \sqrt{\frac{H+{\rm{i}}w}{{c}_{2}}}}erfc\{\frac{\xi }{2\sqrt{{c}_{2}t}}-\sqrt{t(H+{\rm{i}}w)}\}\\  &  & +\,{{\rm{e}}}^{\xi \sqrt{\frac{H+{\rm{i}}w}{{c}_{2}}}}erfc\{\frac{\xi }{2\sqrt{{c}_{2}t}}+\sqrt{t(H+{\rm{i}}w)}\}]\\  &  & -\,\frac{1}{4{\rm{i}}}{{\rm{e}}}^{-itw}[{{\rm{e}}}^{-\xi \sqrt{\frac{H-{\rm{i}}w}{{c}_{2}}}}erfc\{\frac{\xi }{2\sqrt{{c}_{2}t}}-\sqrt{t(H-{\rm{i}}w)}\}\\  &  & +\,{{\rm{e}}}^{\xi \sqrt{\frac{H-{\rm{i}}w}{{c}_{2}}}}erfc\{\frac{\xi }{2\sqrt{{c}_{2}t}}+\sqrt{t(H-{\rm{i}}w)}\}]\end{array},$$

24 $${u}_{b}(\xi ,t)=\frac{1}{2}A\{\begin{array}{c}{{\rm{e}}}^{-\sqrt{-\frac{{c}_{5}}{{c}_{4}}}\xi }(2-erfc[\tfrac{2\sqrt{-{c}_{4}{c}_{5}}t-\xi }{2\sqrt{{c}_{4}t}}]+{{\rm{e}}}^{2\sqrt{-\frac{{c}_{5}}{{c}_{4}}}\xi }erfc[\tfrac{2\sqrt{-{c}_{4}{c}_{5}}t+\xi }{2\sqrt{{c}_{4}t}}])\\ -{{\rm{e}}}^{-\sqrt{\frac{H}{{c}_{2}}}\xi }(2-erfc[\tfrac{2\sqrt{{c}_{2}H}t-\xi }{2\sqrt{{c}_{2}t}}]+{{\rm{e}}}^{2\sqrt{\frac{H}{{c}_{2}}}\xi }erfc[\tfrac{2\sqrt{{c}_{2}H}t+\xi }{2\sqrt{{c}_{2}t}}])\end{array}\},$$

25 $${u}_{c}(\xi ,t)=\tfrac{{B}_{1}{e}^{\tfrac{{c}_{7}}{{c}_{6}}}}{2}\{\begin{array}{c}{e}^{-\xi i\frac{\sqrt{{c}_{5}+\frac{{c}_{7}}{{c}_{6}}}}{\sqrt{{c}_{4}}}}erfc(\tfrac{\xi }{2\sqrt{t}\sqrt{{c}_{4}}}-i\sqrt{({c}_{5}+\tfrac{{c}_{7}}{{c}_{6}})t})+{e}^{\xi i\frac{\sqrt{{c}_{5}+\frac{{c}_{7}}{{c}_{6}}}}{\sqrt{{c}_{4}}}}erfc(\tfrac{\xi }{2\sqrt{t}\sqrt{{c}_{4}}}+i\sqrt{({c}_{5}+\tfrac{{c}_{7}}{{c}_{6}})t})\\ -{e}^{-\xi \frac{\sqrt{H-\frac{{c}_{7}}{{c}_{6}}}}{\sqrt{{c}_{2}}}}erfc(\tfrac{\xi }{2\sqrt{t}\sqrt{{c}_{2}}}-\sqrt{(H-\tfrac{{c}_{7}}{{c}_{6}})t})-{e}^{\xi \frac{\sqrt{H-\frac{{c}_{7}}{{c}_{6}}}}{\sqrt{{c}_{2}}}}erfc(\tfrac{\xi }{2\sqrt{t}\sqrt{{c}_{2}}}+\sqrt{(H-\tfrac{{c}_{7}}{{c}_{6}})t})\end{array}\},$$

26 $$\begin{array}{rcl}{u}_{d}(\xi ,t) & = & \tfrac{\xi C}{2\sqrt{{c}_{4}}\sqrt{\pi }}{\int }_{0}^{t}\{{{\rm{e}}}^{{c}_{5}(t-\tau )}(\tfrac{1}{\sqrt{\pi }\sqrt{t-\tau }}+{c}_{6}{{\rm{e}}}^{{{c}_{6}}^{2}(t-\tau )}erfc[-{c}_{6}\sqrt{t-\tau }])\ast \tfrac{{{\rm{e}}}^{{c}_{5}\tau -\tfrac{{\xi }^{2}}{4{c}_{4}\tau }}}{{\tau }^{3/2}}\}d\tau \\  &  & -\tfrac{\xi C}{2\sqrt{{c}_{2}}\sqrt{\pi }}{\int }_{0}^{t}\{{{\rm{e}}}^{{c}_{5}(t-\tau )}(\tfrac{1}{\sqrt{\pi }\sqrt{t-\tau }}+{c}_{6}{{\rm{e}}}^{{{c}_{6}}^{2}(t-\tau )}erfc[-{c}_{6}\sqrt{t-\tau }])\ast \tfrac{{{\rm{e}}}^{-H\tau -\tfrac{{\xi }^{2}}{4{c}_{2}\tau }}}{{\tau }^{3/2}}\}d\tau .\end{array}$$

## Particular Cases

In order to link our found solutions with published literature, the following particular cases are examined by taking some parameters absent.

Making *Gr* = *γ* = 0 and Re = 1 in Eq. (22), reduces to:

27 $$\begin{array}{rcl}u(\xi ,t) & = & \frac{1}{4{\rm{i}}}{{\rm{e}}}^{{\rm{i}}tw}[{{\rm{e}}}^{-\xi \sqrt{H+{\rm{i}}w}}erfc\{\frac{\xi }{2\sqrt{t}}-\sqrt{t(H+{\rm{i}}w)}\}\\  &  & +\,{{\rm{e}}}^{\xi \sqrt{H+{\rm{i}}w}}erfc\{\frac{\xi }{2\sqrt{t}}+\sqrt{t(H+{\rm{i}}w)}\}]\\  &  & -\,\frac{1}{4{\rm{i}}}{{\rm{e}}}^{-itw}[{{\rm{e}}}^{-\xi \sqrt{H-{\rm{i}}w}}erfc\{\frac{\xi }{2\sqrt{t}}-\sqrt{t(H-{\rm{i}}w)}\}\\  &  & +\,{{\rm{e}}}^{\xi \sqrt{H-{\rm{i}}w}}erfc\{\frac{\xi }{2\sqrt{t}}+\sqrt{t(H-{\rm{i}}w)}\}],\end{array}$$

which is identical to results of ^61^, Eq. (24).

Taking $M=\frac{1}{k}=0$ in the above relation, we get:

28 $$\begin{array}{rcl}u(\xi ,t) & = & \frac{1}{4{\rm{i}}}{{\rm{e}}}^{{\rm{i}}tw}[{{\rm{e}}}^{-\xi \sqrt{{\rm{i}}w}}erfc\{\frac{\xi }{2\sqrt{t}}-\sqrt{{\rm{i}}wt}\}{+e}^{\xi \sqrt{{\rm{i}}w}}erfc\{\frac{\xi }{2\sqrt{t}}+\sqrt{{\rm{i}}wt}\}]\\  &  & -\,\frac{1}{4{\rm{i}}}{{\rm{e}}}^{-itw}[{{\rm{e}}}^{-\xi \sqrt{{\rm{i}}w}}erfc\{\frac{\xi }{2\sqrt{t}}-\sqrt{-{\rm{i}}wt}\}{+e}^{\xi \sqrt{{\rm{i}}w}}erfc\{\frac{\xi }{2\sqrt{t}}+\sqrt{-iwt}\}]\end{array},$$

Which is in accordance with^61^, Eq. (25).

Taking $Gr=M=\frac{1}{k}=\frac{1}{\gamma }=0$, in Eq. (22), it moderates to:

29 $$\begin{array}{rcl}u(\xi ,t) & = & \tfrac{1}{4{\rm{i}}}{{\rm{e}}}^{{\rm{i}}tw}[{{\rm{e}}}^{-\xi \sqrt{{\rm{Rei}}w}}erfc\{\tfrac{\xi \sqrt{{\rm{Re}}}}{2\sqrt{t}}-\sqrt{{\rm{i}}wt}\}{+e}^{\xi \sqrt{{\rm{Rei}}w}}erfc\{\tfrac{\xi \sqrt{{\rm{Re}}}}{2\sqrt{t}}+\sqrt{{\rm{i}}wt}\}]\\  &  & -\,\tfrac{1}{4{\rm{i}}}{{\rm{e}}}^{-itw}[{{\rm{e}}}^{-\xi \sqrt{-\mathrm{Rei}w}}erfc\{\tfrac{\xi \sqrt{{\rm{Re}}}}{2\sqrt{t}}-\sqrt{-{\rm{i}}wt}\}{+e}^{\xi \sqrt{-{\rm{Rei}}w}}erfc\{\tfrac{\xi \sqrt{{\rm{Re}}}}{2\sqrt{t}}+\sqrt{-{\rm{i}}wt}\}]\end{array},$$

Identical to^58^, Eq. (35).

**Skin friction and Nusselt Number**

30 $${C}_{f}=\frac{1}{{(1-\varphi )}^{2.5}}(1+\frac{1}{\gamma }){\frac{\partial u(\xi ,t)}{\partial \xi }|}_{\xi =0},$$

31 $$\begin{array}{rcl}{C}_{f} & = & \frac{1}{2}{{\rm{ie}}}^{-{\rm{i}}tw}\frac{1}{{(1-\varphi )}^{2.5}}(1+\frac{1}{\gamma })\\  &  & \times \,[\begin{array}{c}{1-e}^{2{\rm{i}}tw}-\frac{{{\rm{e}}}^{-t(H-{\rm{i}}w)}}{\sqrt{{c}_{2}t}\sqrt{\pi }}-\sqrt{\frac{H-{\rm{i}}w}{{c}_{2}}}erf[\sqrt{t(H-{\rm{i}}w)}]\\ +{{\rm{e}}}^{2{\rm{i}}tw}\{\frac{{{\rm{e}}}^{-t(H+{\rm{i}}w)}}{\sqrt{{c}_{2}t}\sqrt{\pi }}+\sqrt{\frac{H+{\rm{i}}w}{{c}_{2}}}erf[\sqrt{t(H-{\rm{i}}w)}]\}\end{array}]\\  &  & -\,A\{\frac{{{\rm{e}}}^{-Ht}}{\sqrt{{c}_{2}t}\sqrt{\pi }}+\sqrt{\frac{H}{{c}_{2}}}erfc(\sqrt{Ht})\}\\  &  & -\,{B}_{1}{e}^{\frac{{c}_{7}}{{c}_{6}}}\{\frac{{{\rm{e}}}^{({c}_{5}+\frac{{c}_{7}}{{c}_{6}})t}}{\sqrt{{c}_{4}t}\sqrt{\pi }}+i\frac{\sqrt{{c}_{5}+\frac{{c}_{7}}{{c}_{6}}}}{\sqrt{{c}_{4}}}-\frac{{{\rm{e}}}^{-(H-\frac{{c}_{7}}{{c}_{6}})t}}{\sqrt{{c}_{2}t}\sqrt{\pi }}-\frac{\sqrt{H-\frac{{c}_{7}}{{c}_{6}}}}{\sqrt{{c}_{2}}}\}\\  &  & +\,(\frac{C}{2\sqrt{{c}_{4}}\sqrt{\pi }}-\frac{C}{2\sqrt{{c}_{2}}\sqrt{\pi }})\\  &  & \times \,{\int }_{0}^{t}\{\frac{{{\rm{e}}}^{{c}_{5}(t-\tau )}}{{\tau }^{3/2}}(\frac{1}{\sqrt{\pi }\sqrt{t-\tau }}+{c}_{6}{{\rm{e}}}^{{{c}_{6}}^{2}(t-\tau )}erfc[-{c}_{6}\sqrt{t-\tau }])\}d\tau .\end{array}$$

32 $$Nu=-{\lambda }_{nf}{\frac{\partial \theta (\xi ,t)}{\partial \xi }|}_{\xi =0},$$

33 $$Nu=-\frac{1}{2}{\lambda }_{nf}\lambda \sqrt{{c}_{4}}{\int }_{0}^{t}[\begin{array}{c}{e}^{{c}_{5}(t-\tau )}\{\frac{1}{\sqrt{\pi }\sqrt{t-\tau }}+\lambda \sqrt{{c}_{4}}{e}^{{{c}_{6}}^{2}(t-\tau )}erfc(-\lambda \sqrt{{c}_{4}}\sqrt{t-\tau })\}\\ \ast \{2-erfc(\sqrt{-{c}_{5}\tau })\}\end{array}]d\tau .$$

## Discussion

In this section different parameters including *γ*, *ϕ*, *Gr*, *M*, *K*, Pr, *Nr* Figs 2–11 are plotted. Geometry of problem is shown in Fig. 1. The influence of *γ* on *u*(*y*, *t*) which shows oscillatory behavior increasing first then decreasing is highlighted in Fig. 2.

**Figure 1:**
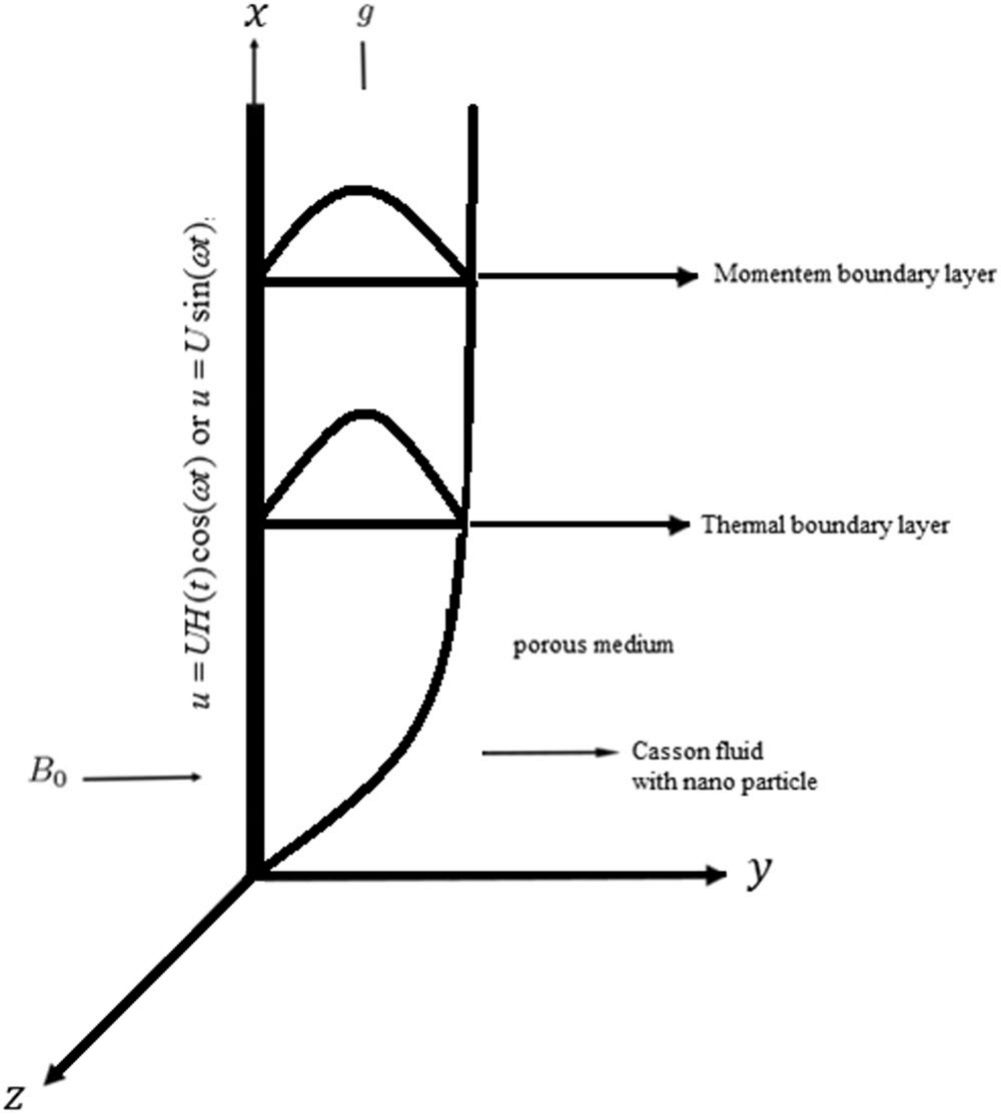
Geometry of the flow.

**Figure 2:**
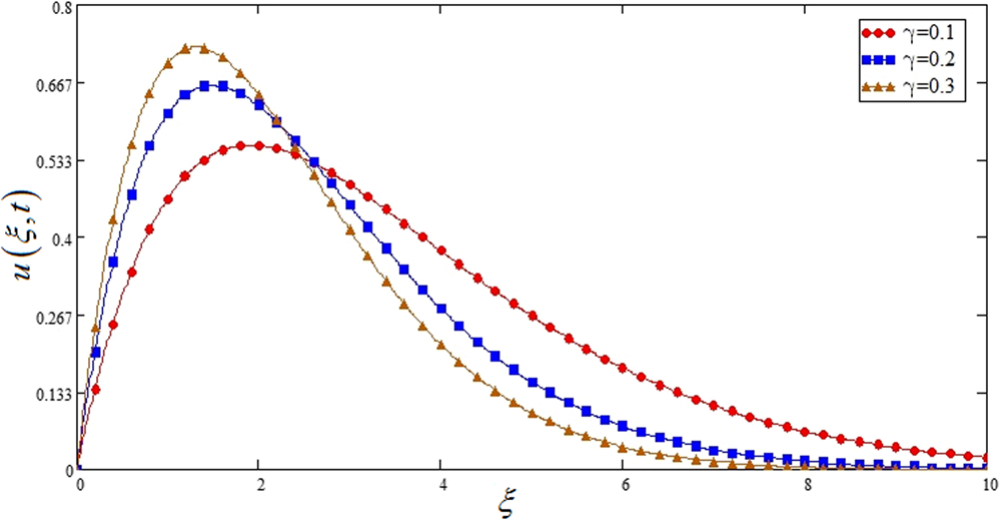
Effects of Casson fluid parameter *γ* on the velocity profile of Sodium alginate based Casson nanofluid when Pr = 0.7, *Gr* = 2 and *φ* = 0.04.

**Figure 3:**
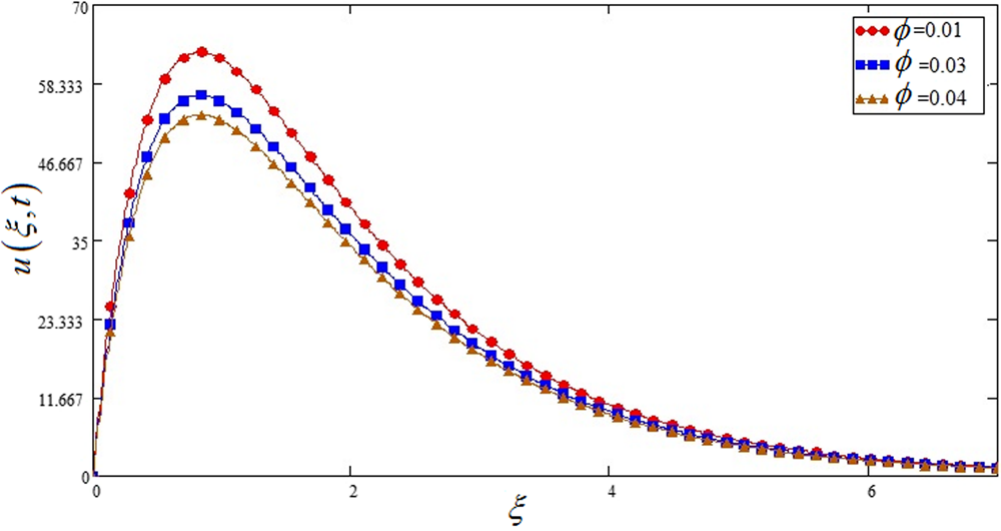
Effects of nano particles volume fraction parameter *φ* on the velocity profile of Sodium alginate based nano fluid when *Gr* = 0.2, *Nr* = 0.2 and *t* = 1.

**Figure 4:**
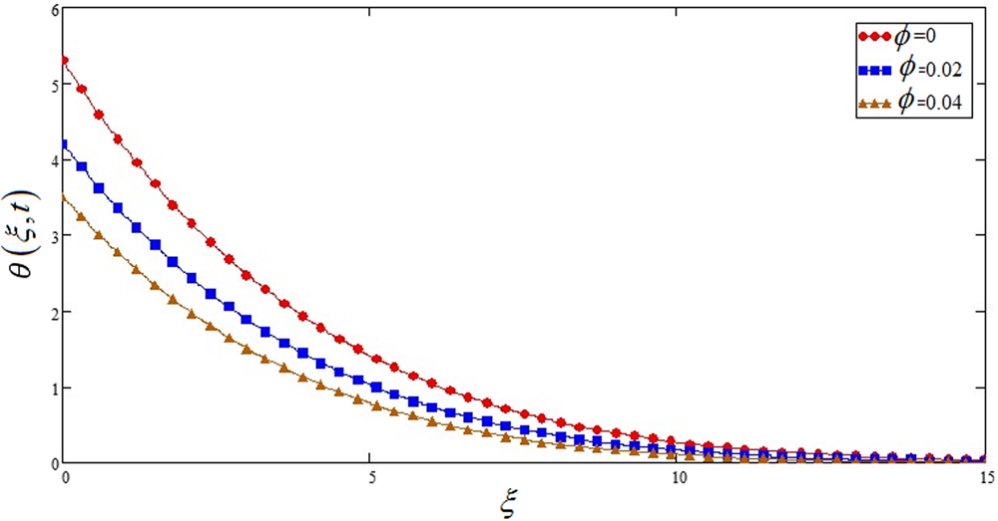
Effects of nano particles volume fraction parameter *φ* on the temperature profile of Sodium alginate based nano fluid when Pr = 5 and *t* = 1.

Figures 3 and 4 show effects of *ϕ* on *u*(*ξ*, *t*) and *θ*(*ξ*, *t*).*φ* is take in between 0 ≤ *ϕ* ≤ 0.04 due to sedimentation when the range goes above from 0.08. It is observed in both cases if the nano particles volume fraction *ϕ* is increased it leads to the decreasing of temperature and velocity profile.

**Figure 5:**
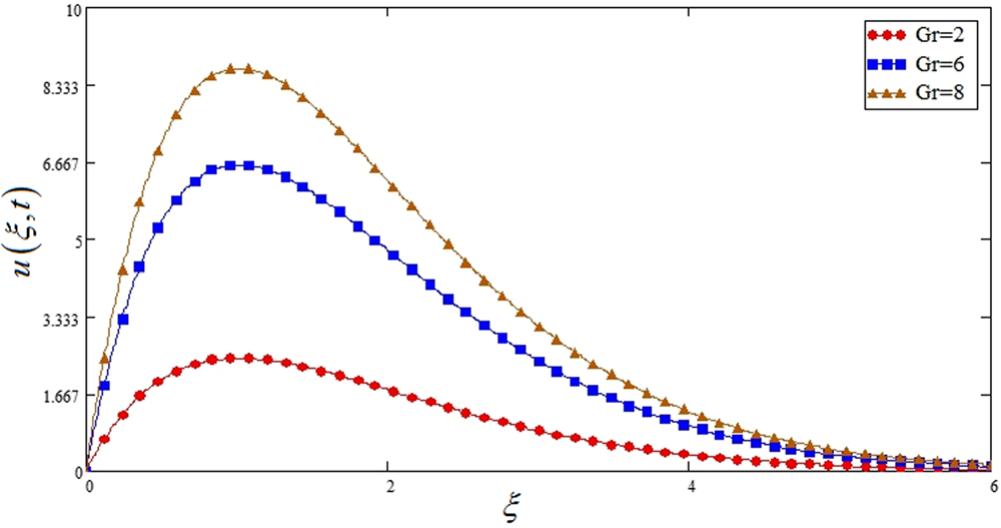
Effects of thermal Grashof number *Gr* on the velocity profile of Sodium alginate based Casson nano fluid when Pr = 0.7, *Nr* = 2, *φ* = 0.04 and *t* = 1.

**Figure 6:**
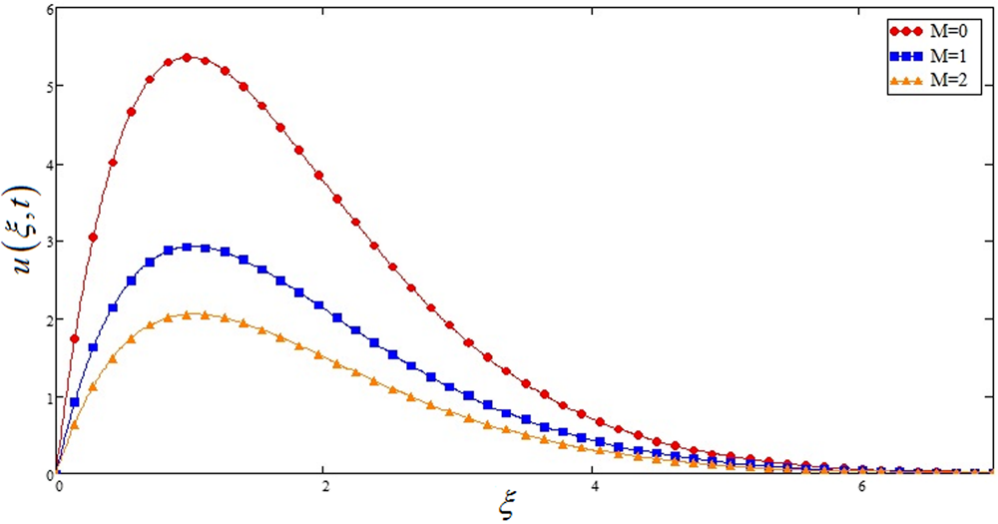
Effects of magnetic parameter *M* on the velocity profile of Sodium alginate based Casson nano fluid when Pr = 0.7, *Nr* = 2, *Gr* = 10, *k* = 2 and *t* = 1.

**Figure 7:**
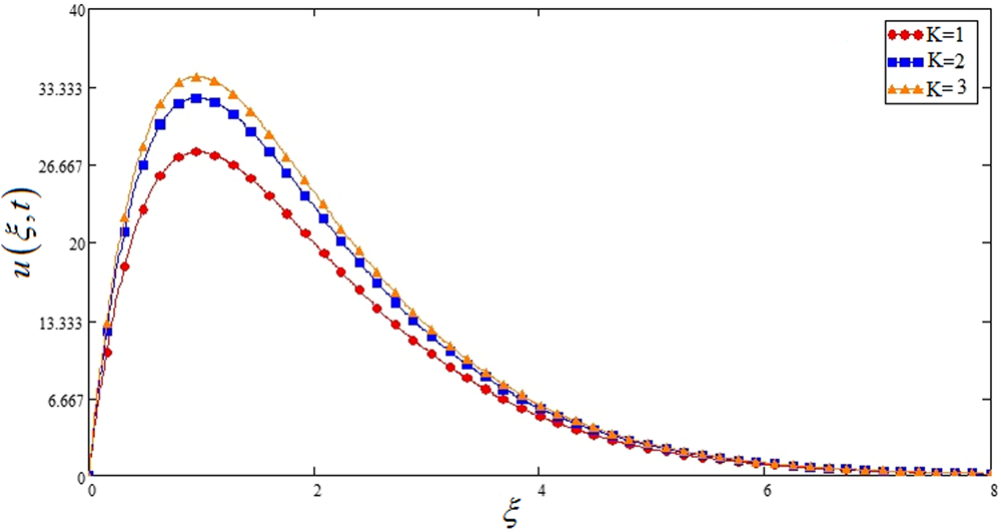
Effects of permeability of porous medium *k* on the velocity profile of Sodium alginate based nano fluid when Pr = 10, *Gr* = 10, *Nr* = 8, *φ* = 0.04 and *t* = 1.

**Figure 8:**
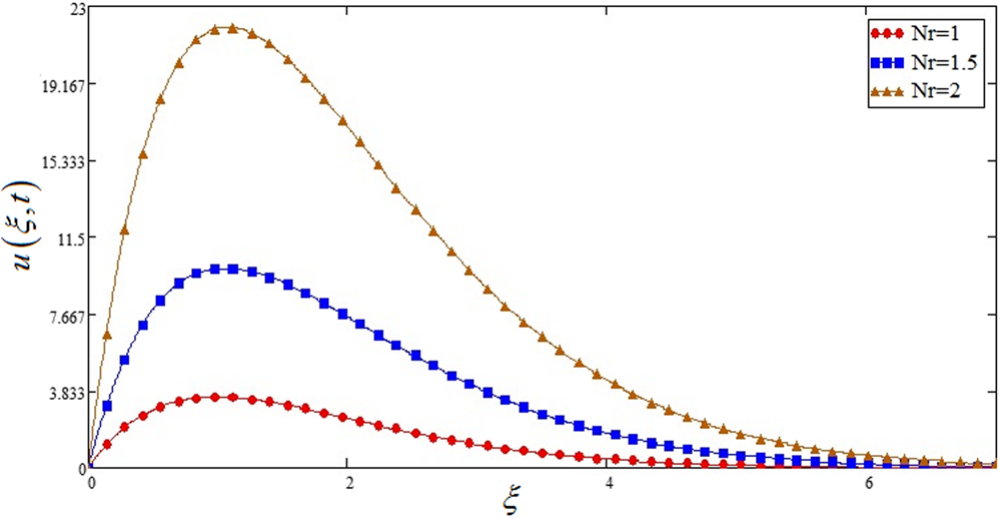
Effects of radiation parameter *Nr* for *TiO*_2_ on the velocity profile of Sodium alginate based nano fluid when Pr = 0.7, *Gr* = 8, *φ* = 0.04 and *t* = 1.

Figure 5 highlights the effect of *Gr* for Sodium alginate -based, Casson nanofluids on velocity profile. It is found that with increasing *Gr*, velocity increases. Because increasing effect in *Gr*, due to increase of buoyancy forces and decrease of viscous forces. Figure 6 the effect of *M* = 0, 1, 2 on the velocity profile. *u*(*ξ*, *t*) decreases due to increasing dragging force. *M* = 0, shows absence of MHD. Figure 7 shows *K* effect of on *u*(*ξ*, *t*). Velocity decrease due to decreasing friction. Figure 8 highlights that profile of velocity is increased with increasing radiation parameter *Nr*. The effect is studied for *TiO*_2_ nano particle.

**Figure 9:**
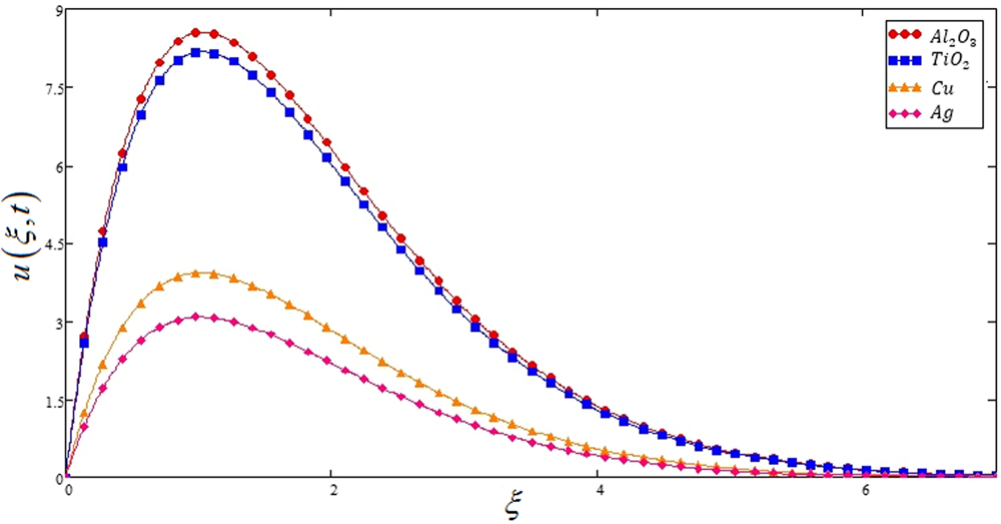
Comparison of velocities profiles for different types of nano particles for Casson nanofluids when Pr = 0.71, *Gr* = 10, *Nr* = 2, *φ* = 0.04 and *t* = 1.

The impact of two different types of nano particles (*Al*_2_*O*_3_ Sodium alginate -based Casson nanofluid and *Ag*-Sodium alginate -based nanofluid) on profile of velocity is studied in Fig. 9. The profile of velocity is greater for *Al*_2_*O*_3_ Sodium alginate -based Casson nanofluid and lower profile velocity for *Ag*-Sodium alginate -based nanofluid is observed.

**Figure 10:**
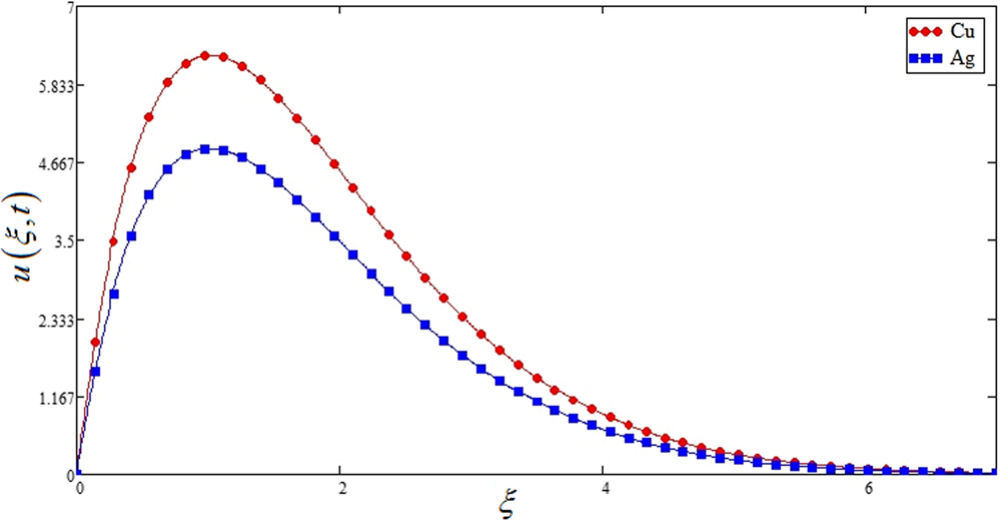
Comparison of velocities profiles of *Cu* and *Ag* Casson nanofluids when Pr = 0.71, *Gr* = 10, *Nr* = 2, *φ* = 0.04 and *t* = 1.

**Figure 11:**
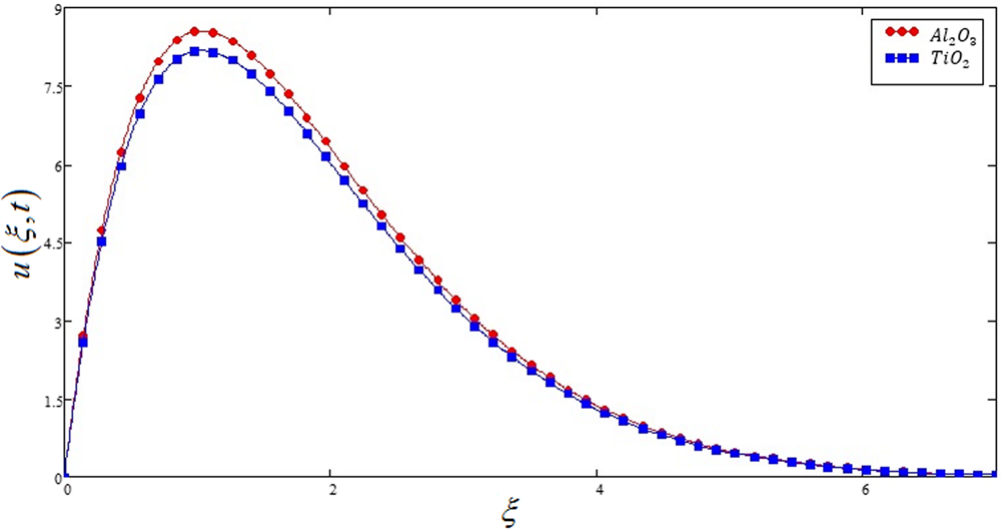
Comparison of velocities profiles of *Al*_2_*O*_3_ and *TiO*_3_ for Casson nanofluids when Pr = 0.71, *Gr* = 10, *Nr* = 2, *φ* = 0.04 and *t* = 1.

Figure 10 highlights the comparison of both (*Cu* Sodium alginate -based Casson nanofluid and *Ag*-Sodium alginate -based nanofluid) on *u*(*ξ*, *t*). Velocity of *Ag*-Sodium alginate -based nanofluid is lower than copper Sodium alginate -based nanofluid. This shows that *Cu* nano particles have more thermal diffusivity compare to *Ag* which is physically true. Furthermore, the same comparison is study for *Al*_2_*O*_3_ and *TiO*_2_ models in Fig. 11, which shows that Aluminum oxide *Al*_2_*O*_3_ nano particles have high thermal diffusivity as compare to Titanium oxide *TiO*_2_.

## Conclusion

The following remarks are concluded from this work:

- *u*(*ξ*, *t*) decreases as *γ* increases
- Temperature and velocity profile are decreasing with increasing nano particles volume
  Fraction *ϕ*.
- *Al*_2_*O*_3_ nanofluid has higher velocity from *TiO*_2_ nanofluid and *Cu* nanofluid has higher velocity from *Ag* nanofluid.
- The pours medium *K* and MHD Μ show opposite behavior.
